# The Distribution of GPR17-Expressing Cells Correlates with White Matter Inflammation Status in Brain Tissues of Multiple Sclerosis Patients

**DOI:** 10.3390/ijms22094574

**Published:** 2021-04-27

**Authors:** Jacopo Angelini, Davide Marangon, Stefano Raffaele, Davide Lecca, Maria P. Abbracchio

**Affiliations:** 1 Dipartimento di Scienze Farmaceutiche, Università degli Studi di Milano, Via Balzaretti 9, 20133 Milano, Italy; jacopoangelini1@gmail.com (J.A.); davide.marangon@unimi.it (D.M.); mariapia.abbracchio@unimi.it (M.P.A.); 2 Dipartimento di Scienze Farmacologiche e Biomolecolari, Università degli Studi di Milano, Via Balzaretti 9, 20133 Milano, Italy; stefano.raffaele@unimi.it

**Keywords:** multiple sclerosis, demyelination, oligodendrocytes, neuropathology

## Abstract

In multiple sclerosis (MS), oligodendrocyte precursor cells (OPCs) are recruited to the site of injury to remyelinate damaged axons; however, in patients this process is often ineffective due to defects in OPC maturation. The membrane receptor GPR17 timely regulates the early stages of OPC differentiation; however, after reaching its highest levels in immature oligodendrocytes, it has to be downregulated to allow terminal maturation. Since, in several animal models of disease GPR17 is upregulated, the aim of this work was to characterize GPR17 alterations in MS patients. We developed immunohistochemistry and immunofluorescence procedures for the detection of GPR17 in human tissues and stained post-mortem MS brain lesions from patients with secondary progressive MS and control subjects. The inflammatory activity in each lesion was evaluated by immunohistochemistry for the myelin protein MOG and the HLA antigen to classify them as active, chronic inactive or chronic active. Hence, we assessed the distribution of GPR17-positive cells in these lesions compared to normal appearing white matter (NAWM) and white matter (WM) of control subjects. Our data have shown a marked increase of GPR17-expressing oligodendroglial cells accumulating at NAWM, in which moderate inflammation was also found. Furthermore, we identified two distinct subpopulations of GPR17-expressing oligodendroglial cells, characterized by either ramified or rounded morphology, that differently populate the WM of healthy controls and MS patients. We concluded that the coordinated presence of GPR17 in OPCs at the lesion sites and inflamed NAWM areas suggests that GPR17 could be exploited to support endogenous remyelination through advanced pharmacological approaches.

## 1. Introduction

Oligodendrocyte precursor cells (OPCs) participate to remyelination under both physiological and pathological conditions by differentiating to mature, myelin producing cells. In multiple sclerosis (MS), this process is often impaired, resulting in blockade of oligodendroglial differentiation and inadequate myelin repair, with consequent alterations of impulse transmission, axonal damage, and neurodegeneration [1]. The reasons at the basis of impaired remyelination in MS are still largely unknown. Despite a recent study indicated that the oligodendroglial differentiation block is not due to intrinsic oligodendroglial factors, but rather caused by the inflammatory environment inside the lesions [2], anti-inflammatory drugs alone are not sufficient to bypass this block, suggesting that their combination with pro-remyelinating agents may represent the road to the therapeutic success. To address this issue, various molecular pathways regulating OPC maturation are under investigation, especially for the progressive forms [3]. In the last 10 years, the oligodendroglial G protein-coupled receptor GPR17 has been progressively demonstrated to play a key role in oligodendrocyte maturation and to ‘label’ a subset of OPCs specifically involved in reaction to injury [4,5]. GPR17 is needed for the first phases of OPC differentiation but has to be downregulated in immature OPCs immediately before terminal maturation [6,7]. Any alteration in this peculiar expression pattern results in myelination defects [8,9,10].

In vitro, persistent GPR17 overexpression in OPCs indeed leads to blockade of cells at immature stages [10]. Interestingly, pathologically increased levels of GPR17 have been found in several animal models of disease, including focal demyelination [11], brain ischemia [6,12], amyotrophic lateral sclerosis [13], and traumatic brain injury [11], suggesting that aberrant GPR17 upregulation may contribute to remyelination failure. In particular, the subset of GPR17-expressing OPCs has been specifically associated to rapid responses to myelin injury, regardless the type of insult (i.e., after ischemic, traumatic, or toxic injuries [4,10,14,15]). These data suggest that receptor expression confers the ability to this cell subset to immediately react and undergo differentiation, to generate fully mature, myelin producing oligodendrocytes repairing the lesion. Regarding the factors leading to GPR17 dysregulation in neurological disorders, increasing evidence in different models of demyelination suggests that GPR17 overexpression is sustained by pro-inflammatory cytokines accumulating close to inflamed lesions [14]. Among these, the stromal derived factor 1 (SDF1) can specifically activate GPR17 [16], leading to an aberrant potentiation of its signaling.

Due to the localization of GPR17 on the membrane of highly responsive precursor cells at the lesion sites, GPR17 could represent a suitable target for pharmacological interventions aiming at removing this blockade and restore myelin repair [4,6,11].

To date, only few independent studies have investigated the expression of GPR17 in human specimens. The most relevant data showed that, after traumatic brain injury, GPR17 is expressed by OPCs with a decreasing gradient starting from the center of lesion [17], whereas in MS an increased level of GPR17 transcript has been detected in active lesions compared to normal appearing white matter (NAWM) [8]. Interestingly, recent data coming from single-nucleus RNA sequencing of post-mortem white matter samples from human brain of MS patients and unaffected controls indicated that GPR17 expression is restricted to specific oligodendroglial subclusters, namely OPCs and committed oligodendrocyte precursors, although these results are not sufficient to compare the receptor levels in MS lesions and in the NAWM with healthy tissues [18].

Here, for the first time, we focused on human brain tissues of MS subjects, in order to explore possible links between GPR17 protein expression and specific histopathological conditions (active, chronic active and chronic inactive lesions, NAWM of MS patients and normal white matter (NWM) of healthy controls), with the ultimate goal to propose GPR17 as a new exploitable target to foster endogenous remyelination.

## 2. Results

### 2.1. All the Histopathological Conditions of Human Brain MS Lesions Can Coexist and They Can Be Disseminated both in Grey and White Matter

In samples deriving from patients with MS, we identified a total of 69 demyelinating lesions: 26 in white matter (WM) and 43 in grey matter. In the present study, we analyzed only WM lesions, namely 16 Active Lesions (ALs), 3 Chronic Active Lesions (CALs), and 7 Chronic Inactive Lesions (CILs). In all samples deriving from patients with diagnosis of MS, we also analyzed the areas of normal appearing WM with no demyelination (NAWMs) and compared them with the normal WM of control subjects (NWMs). Consistently, no demyelinating lesions were found in controls. Representative images of these different histopathological conditions are shown in Figure S1.

### 2.2. GPR17^+^ Cells Are More Represented in Inflamed White Matter of MS Patients and They Belong to Early Oligodendroglial Lineage

**Figure 1:**
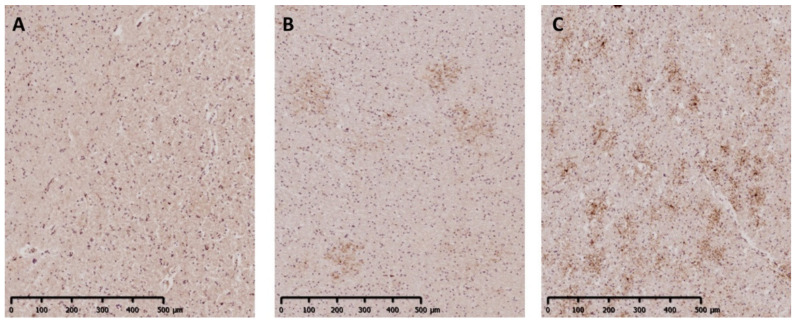
Evaluation of the abundance of GPR17^+^ cells. The representative micrographs show tissues with different abundance of the receptor: panel (**A**) (sample C 036-A1B2): absence (−); panel (**B**) (MS 286-P2A3): low abundance (+); panel (**C**) (MS 234-A2D4): high abundance (++). This scoring system has been used for the qualitative classification reported below. Scale bar 500 µm.

To characterize the expression of GPR17 in human lesions and to evaluate a possible correlation among groups, we performed immunohistochemistry analyses on all samples from patients and controls. All the conditions (AL/CAL/CIL/NAWM/NWM) from cases and controls were scored according to a qualitative evaluation of the amount of GPR17^+^ cells (Figure 1). As shown in Table 1, we found that GPR17^+^ cells were widespread in all the analyzed tissues and that NAWM regions of MS subjects were particularly enriched in GPR17^+^ cells compared to the lesion areas and NWM in control subjects.

**Figure 2:**
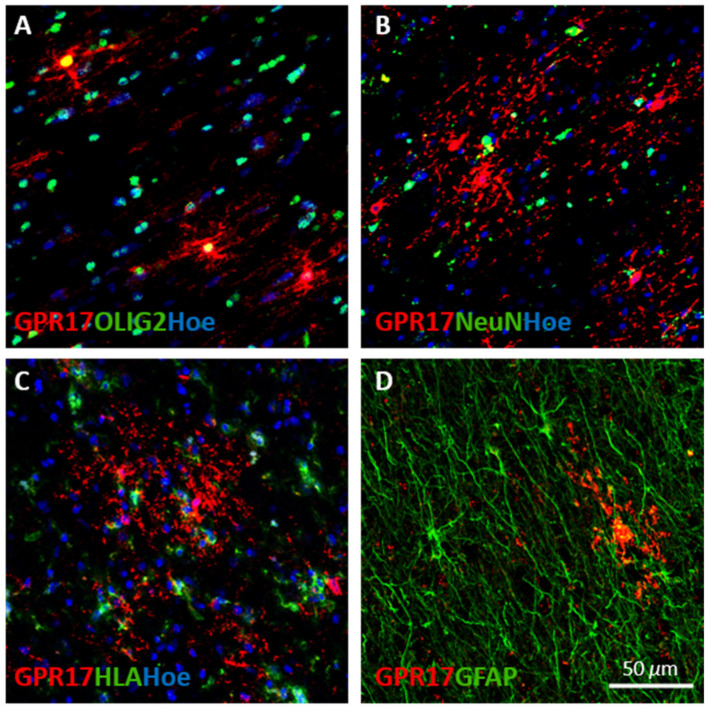
Identification of GPR17^+^ cells in human brain white matter. The confocal micrographs show the co-localization of GPR17 (in red) with the specific lineage markers for (**A**) oligodendrocytes (Olig2), (**B**) neurons (NeuN), (**C**) inflammatory cells (HLA), and (**D**) astrocytes (GFAP), in green. Cell nuclei were labelled with Hoechst 33258 (Hoe), in blue. Scale bar 50 µm.

To univocally identify the phenotype of GPR17^+^ cells, we performed double immunofluorescence staining for specific lineage markers, namely Olig2 for oligodendroglial cells, NeuN for neurons, HLA for inflammatory immune cells, and GFAP for astrocytes. As shown in Figure 2, all the GPR17-expressing cells colocalized with Olig2, regardless of their morphology, whereas no colocalization was found with NeuN, HLA, and GFAP, thus confirming the exclusive oligodendroglial nature of stained cells.

**Figure 3:**
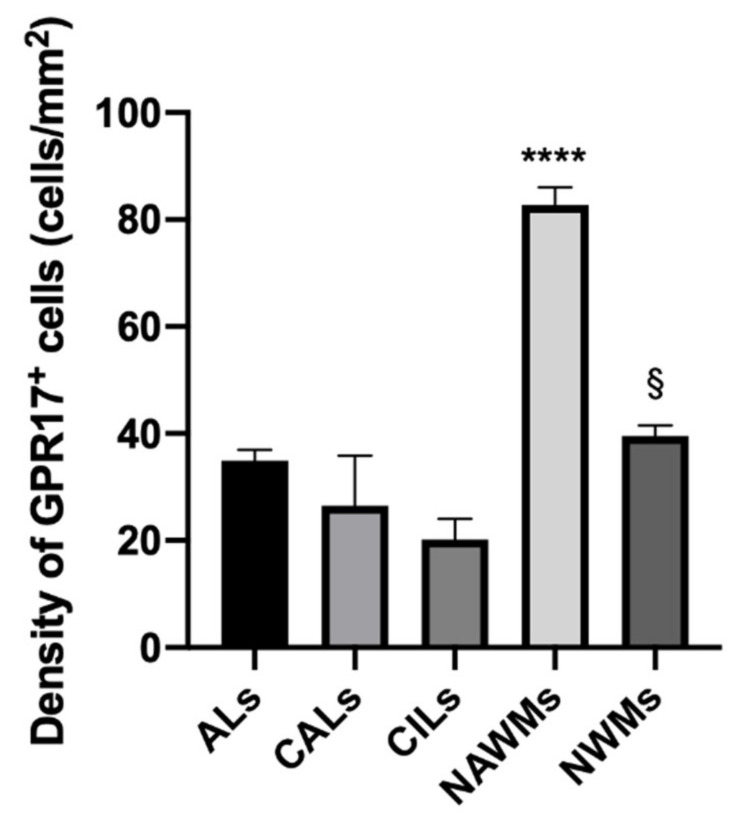
Quantification of GPR17^+^ cells density in white matters of brain tissues in MS patients and healthy controls. The density of GPR17^+^ cells was calculated as number of marked cells per mm^2^ of analyzed area. The GPR17^+^ cells populate all the conditions, as the follows (mean ± SD). ALs = 34.99 ± 3.98; CALs = 26.51 ± 13.2; CILs = 20.2 ± 7.66; NAWMs = 82.67 ± 6.73; NWMs = 39.55 ± 3.43. According to a comparative analysis, a higher prevalence of GPR17^+^ cells was demonstrated in NAWMs than in all the other conditions, (**** *p*-value ≤ 0.0001). Furthermore, GPR17 was more represented in NWMs than CILs (§ *p*-value= 0.02). ANOVA one-way test. ALs: active lesions; CALs: chronic active lesions; CILs: chronic inactive lesions; NAWMs: normal appearing white matter in patients with multiple sclerosis; NWMs: normal white matter in healthy controls.

Finally, we performed a quantitative analysis of GPR17^+^ cell density in four random samples per each condition. According to the descriptive analysis, the density of GPR17^+^ cells was significantly increased in NAWMs of MS patients compared to all other conditions (Figure 3), suggesting that this OPC population reacted to the nearby damage starting to differentiate, but then failed to reach the lesion sites and participate to remyelination. We did not observe significant differences in the density of GPR17^+^ cells comparing ALs, CALs, and NWMs to each other. Instead, we found a significant reduction of GPR17^+^ cells in CILs compared to NWMs (mean ± SD, NWMs = 39.55 ± 3.43 vs. CILs = 20.2 ± 7.66 cells/mm^2^) (Figure 3).

### 2.3. GPR17^+^ Cells Show Two Different Morphologies Which Are Associeted with Specific Histological Conditions

**Figure 4:**
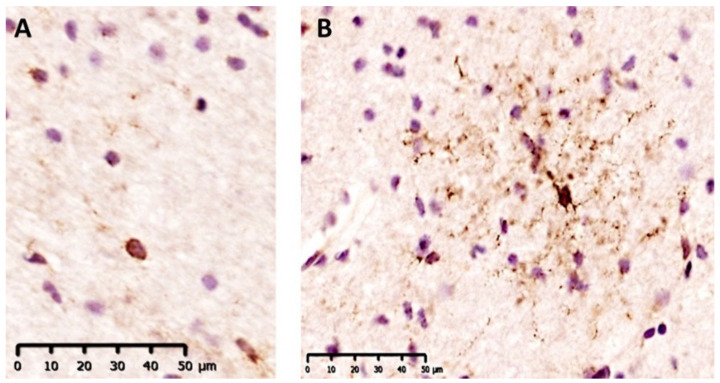
Representative pictures of GPR17^+^ cells (brown staining) with rounded (**A**) and ramified morphology (**B**). Nuclei were counterstained with hematoxylin (in purple). Scale bar 50 µm.

We observed that our GPR17 antibody decorates two distinct populations of positive cells, with a clearly distinguishable morphology: in the former, only cell bodies were stained (rounded morphology), whereas in the latter both cell bodies and fine ramifications were labeled (ramified morphology) (Figure 4).

To evaluate whether the two types of GPR17-expressing subpopulations were preferentially associated to a specific condition, we separately analyzed ramified and rounded GPR17^+^ cells in all the samples analyzed.

**Figure 5:**
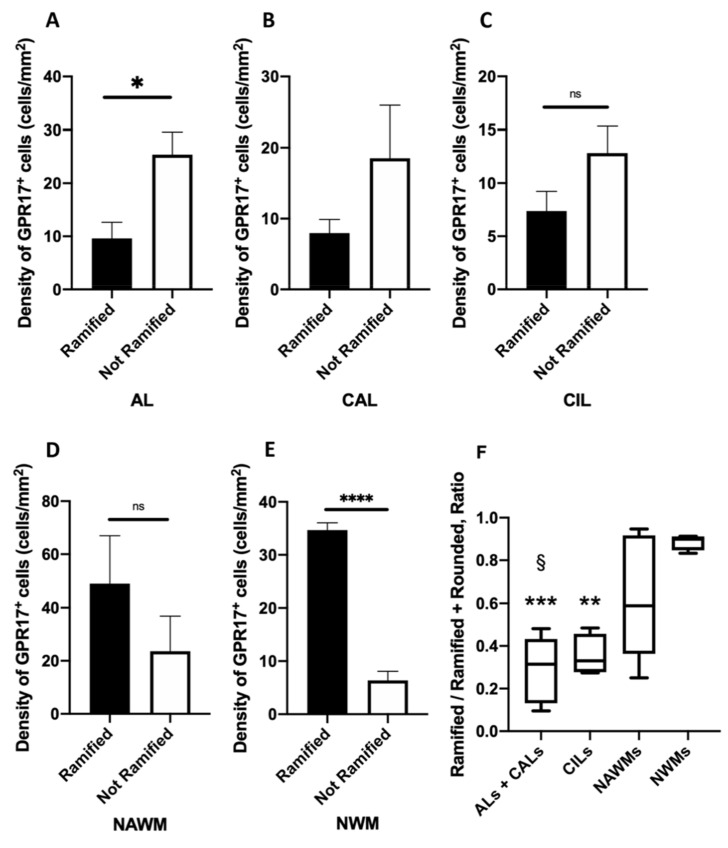
Density and morphology of GPR17^+^ cells in human white matter. The histograms (**A**–**E**) highlight the number of GPR17^+^ cells per area in each condition (ALs, CALs, CILs, NAWMs, and NWMs) based on morphology (ramified or rounded). Data are the mean ± SEM. Unpaired Student’s *t*-test. * *p*-value < 0.05; **** *p*-value < 0.0001. (**F**) The prevalence of ramified vs. total of GPR17^+^ cells in active (ALs + CALs), inactive (CILs) lesion, NAWMs and NWMs is shown. In the *Y* axis the ratio between ramified GPR17^+^ cells and the sum of ramified and rounded GPR17^+^ cells of each condition. Comparative analysis demonstrates that the ramified GPR17^+^ cells are more represented in NWMs than in active lesions (ALs + CALs vs. NWMs; *** *p*-value = 0.0007), and CILs (CILs vs. NWMs; ** *p*-value < 0.005), and are more represented in NAWM than in active lesions (ALs + CALs vs. NAWMs; § *p*-value < 0.05). Data are presented as the mean ± SEM. ANOVA one-way test. ALs: active lesions; CALs: chronic active lesions; CILs: chronic inactive lesions; NAWMs: normal appearing white matter in patients with multiple sclerosis; NWMs: normal white matter in healthy controls.

First, we assessed the prevalence of a specific population in ALs, CALs, CILs, NAWMs, and NWMs, separately. A higher prevalence of rounded GPR17^+^ cells was found in ALs, whereas, in NWMs the ramified morphology was the most represented (Figure 5A,E). No remarkable differences between the two subpopulations of GPR17^+^ cells were found in CALs, CILs, and NAWMs (Figure 5B–D).

Then, we performed a comparative analysis among active lesions (ALs and CALs), CILs, NAWMs, and NWMs, in order to evaluate the correlation between a specific condition and the prevalence of ramified or rounded GPR17^+^ cells. For this reason, we calculated the ratio between ramified GPR17^+^ cells and the total GPR17^+^ cells in each condition and found that the ramified GPR17^+^ cells are more represented in NWMs than in active lesions or CILs and are more represented in NAWMs than in active lesions, whereas in all the other conditions no remarkable differences were found (Figure 5F).

### 2.4. Rounded GPR17^+^ Cells Specifically Populate the Core of Acute Lesions

To investigate whether a spatial gradient of GPR17^+^ cells was present in the lesions, we separately analyzed the lesion cores and their delimiting borders, considering as ‘border area’ the WM located within 200 µm from the perimeter of the demyelinated lesion (Figure S2).

**Figure 6:**
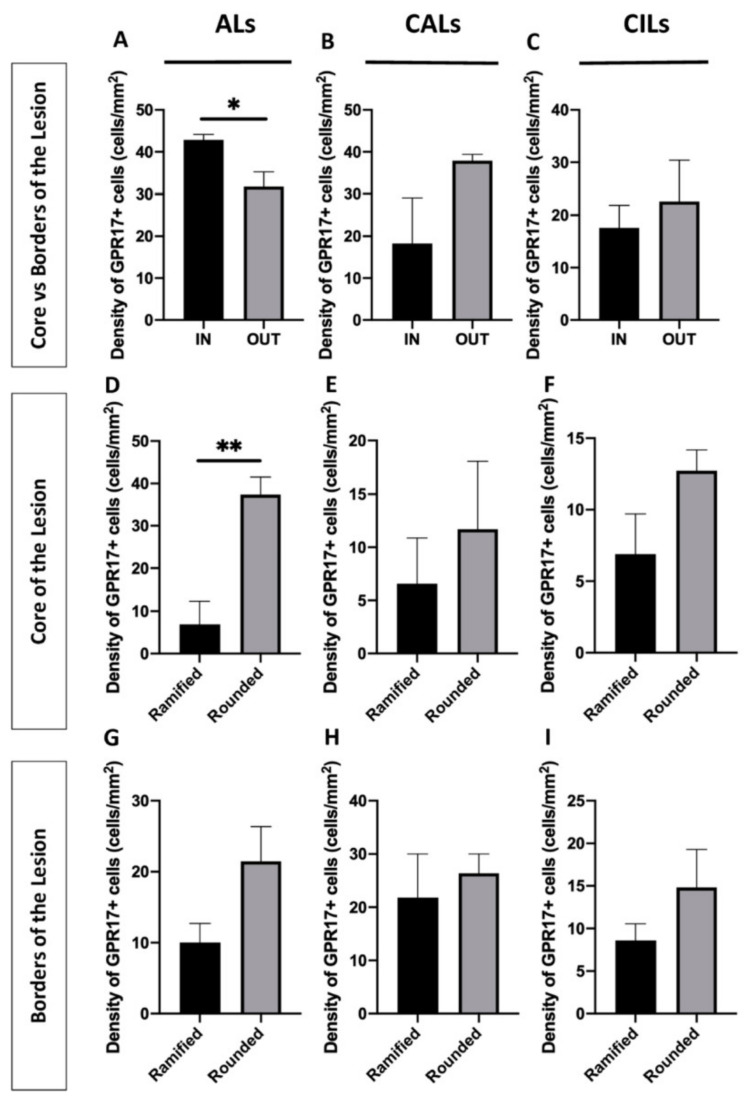
Analysis of GPR17^+^ cells distribution and morphology in MS lesions. In ALs (**A**) the overall GPR17^+^ cells are more localized inside the lesion (* *p*-value < 0.05), whereas no difference in terms of distribution inside/outside was found for the other lesions (**B**,**C**). In (**D**–**I**), the histograms show the distribution of ramified and rounded GPR17^+^ cells according to their localization, inside (**D**–**F**) and at the borders (**G**–**I**), in each kind of lesion. The rounded morphology was significantly overrepresented into the AL core compared to the ramified one (** *p*-value < 0.005). Unpaired Student’s *t*-test. ALs: active lesions; CALs: chronic active lesions; CILs: chronic inactive lesions; IN: inside the lesion; OUT: at the border of the lesion.

First, we compared the density of GPR17^+^ cells into the core of the lesion with those gathered at the border in each condition. We observed that, in ALs, GPR17^+^ cells were more abundant inside the lesion, whereas in CALs and CILs no differences were found (Figure 6A–C).

Then, we looked for the most represented morphology of GPR17^+^ cells at the border and in the core of ALs, CALs, and CILs. No prevalence of a particular morphology of GPR17^+^ cells were detected inside/outside the different lesions, except for ALs, where rounded GPR17^+^ cells outnumbered ramified ones inside the lesion. No differences were detected outside (Figure 6D–I).

## 3. Discussion

In the last 10 years, GPR17 has progressively emerged as a key player in the physiological differentiation of adult OPCs [4,5] and several pre-clinical studies have specifically associated its expression to rapid responses to myelin injury, independently of the type of insult (i.e., after either ischemic, traumatic, or toxic injuries [4,10,14]. Despite these results suggesting GPR17 as a new potential target for remyelination therapies in MS, to date, expression and spatial distribution of the GPR17 receptor in MS tissues has not been fully investigated.

Our data show that in both control subjects and patients, the receptor is expressed exclusively by oligodendroglial cells, supporting the involvement of GPR17 not only in diseases but also in the physiological maturation of human oligodendrocytes [9]. Both qualitative and quantitative analyses consistently show that GPR17^+^ cells are widespread in all samples; however, notably, the NAWM clearly presents the highest amount of GPR17 expressing cells. Apparently in contrast with this finding, Chen et al. previously suggested an increase of GPR17 expression in MS plaques compared to NAWM [8]. However, the two studies are not directly comparable: (a) in Chen et al., GPR17 expression was only evaluated at transcriptional level and in the whole tissue, without considering the effects of post-transcriptional mechanisms (e.g., miRNAs, RNA binding protein, RNA editing) and the number of the expressing cells. Instead, our analysis refers to GPR17 protein and GPR17-expressing cells in specific areas; (b) it was a bulk analysis in which MS plaques were not characterized according to the histological classification [19], while the present data show that pre-active, active, inactive, chronic and acute lesions are characterized by highly different expression levels of GPR17; (c) the Chen et al. analysis did not consider the anatomy of the lesions, whereas in the present study we have excluded the peri-plaque areas of NAWM, which we observed to be characterized by highly remarkable GPR17 positivity.

In line with our observations, data from literature have shown that NAWM is characterized by relevant and diffuse ongoing inflammation, in the absence of demyelination [20,21,22,23,24]. The abundance of GPR17^+^ cells in this area suggests that GPR17 expression is not related to a specific kind of demyelinating lesion, but rather to tissue responsivity and inflammatory status. We cannot exclude that the abundance of GPR17^+^ cells may be the consequence of an increased OPC proliferation and subsequent differentiation attempt. Previous findings have shown that the receptor is activated by different inflammatory mediators [6,16,25,26] and that there is a positive correlation between the density of OPCs and macrophages in MS patients [27]. Thus, the analysis of NAWM may be particularly relevant to understand how lesions originate and eventually to investigate new mechanisms to prevent demyelination in the very early phases of the disease.

On top of this, our immunohistochemistry and immunofluorescence analysis confirmed the coexistence of two morphologically distinguishable types of oligodendroglial GPR17-expressing cells—i.e., ramified and rounded cells—which could represent different OPC maturation stages. In this respect, it is known that, in rodent species, GPR17 starts to be expressed in the soma of early quiescent OPCs characterized by a simple rounded morphology typical of undifferentiated cells. OPCs can also show a rounded morphology when, upon activation, they migrate in response to lesion context [28,29,30], or are exposed to prolonged metabolic stress induced by inflammation, which is characterized by a decrease of processes ramifications [31]. During maturation, OPCs start extending cellular processes and, at the same time, the expression of GPR17 progressively increases, thus also in humans, ramified GPR17^+^ cells may represent resident pre-oligodendrocytes undergoing maturation. However, double immunofluorescence staining with specific markers of OPC differentiation stages will be necessary to confirm this hypothesis. In the NAWMs of our samples, ramified and rounded morphologies were equally represented. The coexistence of both GPR17^+^ cells morphologies could reflect different coexisting physio-pathological states in NAWM [32], which is a dynamic and reactive tissue where unknown conditions can eventually trigger an AL. The persistent pro-inflammatory milieu in NAWM could block oligodendrocytes in an immature stage and force a prolonged and untimely expression of GPR17. We postulate that this overexpression is partly responsible of remyelination impairment, which may have relevant implications for the treatment of MS.

When inflammation remarkably increases in NAWM, infiltrating T-lymphocytes, activated microglia and autoantibodies against myelin induce demyelination and further infiltration in the core of the rising active lesion with widespread cytotoxic effects against resident cells. For this reason, ALs are hypocellular, with a low number of spread OPCs expressing GPR17, in contrast to NAWM, where the tissue is still intact and a relevant amount of GPR17+ cells is found, reflecting their significant response in order to remyelinate. However, this recruitment is not sufficient in ALs, likely due to limited migration capabilities of OPCs surviving the inflammatory milieu of NAWM [33,34,35]. When inflammation goes down, the lesion appears demyelinated and even more hypocellular, with very low presence of inflammatory cells and oligodendrocytes due to advanced cell loss (CILs). Consistently, our data show a low amount of GPR17^+^ cells in CILs, due to overall loss of the original anatomical architecture and the less responsive milieu of inactive lesions than the WM in physiological condition [32].

In the present study, the difference of age between control cases and patient groups (73.8 vs. 53.8 years old, respectively) may have a potential impact on our results. Indeed, it was known that aged brain tissues are physio-pathologically characterized by increased inflammation compared to younger ones, even in the absence of disease [36,37]. However, since all data suggest a positive correlation between GPR17 expression and inflammation, it is likely that the analysis of age-matched groups may extend the significant difference that we have already observed between the NAWM of patients and the NWM of controls. The control group also showed a gender unbalance (1 female vs. 4 males), but the numerosity is too low to make any statements about the impact on the analyzed outcomes.

Considering GPR17 distribution in MS brain tissues, its strong correlation with inflammation levels in WM and the direct involvement in myelin genesis and repair, we confirm this receptor as a promising target for pro-remyelinating therapies, which today still represent an unmet medical need in MS treatment. As a membrane G protein-coupled receptor, GPR17 is easily druggable. Moreover, its expression in highly reactive cells close to the lesion sites, next to the blood–brain barrier, which is more permeable in MS patients, could facilitate the interaction of the putative pro-remyelinating compound and GPR17. On this basis, we propose that innovative and selective ligands for GPR17, in combination with other agents acting on inflammation and/or myelination inhibitors (e.g., LINGO-1, CSPGs, BMPs), could restore the adequate physiological maturation of oligodendrocytes and their myelinating properties.

From the pharmacodynamic point of view, both the agonist galinex [38] and antagonist—as cangrelor, montelukast, or pranlukast—showed promising results in preliminary in vivo studies [6,12,26,38,39,40,41,42]. Nevertheless, antagonist ligands could potentially cause a total arrest of the GPR17 physiological signaling in healthy tissue and lead to safety issues in case of the long-term treatments. In this respect, we hypothesize that the best option may be the use of a partial agonist, which should both preserve the physiological function of GPR17 where needed and prevent its pathological overexpression in injured areas.

## 4. Materials and Methods

### 4.1. Sample Acquisition

All the analyses described were performed on brain autoptic samples and pertinent donors’ clinical records provided by the UK Multiple Sclerosis Tissue Bank of the Imperial College of London. All patients and the next of kin had given written consent for autopsy and for use of their brain tissue for scientific research, with full ethical approval by UK Ethics Committee (ref. no. 08/MRE09/31).

Cerebral tissues from nine patients with diagnosis of MS (three men and six women) have been compared with other ones deriving from five patients without diagnosis of central nervous system demyelinating diseases (four men and one woman). Clinical and demographic characteristics are shown in Table 2.

### 4.2. Immunohistochemistry on Human Tissues

Snap frozen autoptic blocks were stored at −80 °C. 12 µm-thick coronal sections were cut in cryostat. Slides were removed from cryostorage (−80 °C) and were washed for 15 min with a phosphate buffered saline solution 1X (PBS 1X) (Lonza, Basel, Switzerland) at room temperature. Then they were incubated in a blocking and fixing solution, containing 0.1% H_2_O_2_ in methanol (Sigma-Aldrich, Milano, Italy), for 20 min at −20 °C. After a second washing in PBS 1X for 15 min at room temperature, brain tissues were incubated with a blocking solution, containing 0.1% Triton and 10% Normal Goat Serum (NGS) (Sigma-Aldrich, Italy) in PBS 1X, for 1 h at room temperature. Then, sections were incubated with primary antibodies overnight at 4 °C in PBS 1X with 0.1% Triton and 5% NGS for all the studied antigens, except for GPR17 which needed 10% NGS. The primary antibodies used are the following: rabbit α-MOG (Proteintech, Manchester, UK) (1:200), mouse α-HLA (Agilent Technologies, Milan, Italy) (1:200) and rabbit α-GPR17 (custom-made by Primm, Naples, Italy) (1:400). After primary antibody incubation, the sections were washed in PBS 1X for 15 min and incubated with an appropriate kit, containing biotinylated secondary antibody (ImmPRESS HRP Reagent KIT—α-rabbit or α-mouse, depending on the species of primary antibody; Vector Laboratories, Burlingame, CA, USA) for 1 h at room temperature. Tissue was stained with 3,3′-diaminobenzidine (DAB, Sigma-Aldrich, Italy) and counterstained with hematoxylin (Sigma-Aldrich, Italy). After dehydration, the slides were mounted with DPX mounting medium (Sigma-Aldrich, Italy).

The images were acquired at the optical microscope by means of NanoZoomer S60 Digital Slide Scanner C13210-01 (Hamamatsu) at NOLIMITS, an advanced imaging facility established by Università degli Studi di Milano, and elaborated with the NDP.view2 software (Hamamatsu Photonics, Hamamatsu, Japan).

### 4.3. Immunofluorescence on Human Tissues

The slides were removed from cryostorage (−80 °C) and were washed for 5 min with PBS 1X at room temperature. Then they were incubated in a blocking and fixing solution composed of 0.1% H_2_O_2_ in methanol for 20 min at −20 °C. After a second washing in PBS 1X for 15 min at room temperature, brain tissues were incubated with a blocking solution with 10% NGS in PBS 1X for 45 min at room temperature. In case of Olig2 staining, the blocking step was performed with 1% BSA in PBS 1X-Tween 0.05% for 1 h at room temperature. These sections were double-labeled with rabbit α-GPR17 primary antibody (1:300) and another of the following: mouse α-NeuN (1:100; Merck Millipore, Milano, Italy) or mouse α-HLA (1:100; Dako, Bolzano, Italy) or goat α-Olig2 (1:200; Bio-techne, Milan, Italy) or mouse α-GFAP (1:400; Merck Millipore, Italy). All primary antibodies were diluted in 10% NGS in PBS 1X and incubated overnight at room temperature, except α-Olig2 which required 1% BSA in PBS 1X-Tween 0.05%. Following primary antibody incubation, the sections were washed and incubated with fluorescent secondary antibodies Alexa 555 or 488 (Life Technologies, Monza, Italy), diluted in 5% NGS in PBS 1X (1:400 for GPR17 secondary labeling, 1:100 for HLA and NeuN secondary labelling, 1:800 for GFAP secondary labelling), for 1 h at room temperature. Only for Olig2 staining, the sections were incubated for 2 h at room temperature with anti-goat secondary antibody (1:500) diluted in 1% BSA in PBS 1X-Tween 0.05%. Hoechst 33258 (Sigma-Aldrich, Italy) was used to visualize cell nuclei. After processing, sections were mounted on microscope slides with fluorescent mounting medium (Dako, Italy). The sections were analyzed by confocal microscope (Nikon A1, Italy).

### 4.4. Classification of Demyelinating Lesions

Immunohistochemistry analysis was used to identify all the required histopathological features for the classification of MS lesions according to previously published method [19,43,44,45]. MOG and HLA immunostainings were performed on consecutive sections for each sample in order to identify active lesions (ALs), chronic active lesions (CALs), and chronic inactive lesions (CILs) [19]. All the MS lesions are characterized by demyelination (absence of MOG immunostaining) and their inflammatory activity is represented by the density of HLA^+^ cells: in ALs inflammatory cells are widespread in all the lesion area, in CALs they are gathered at the lesion borders only, whereas in CILs a very low number of HLA^+^ cells is detected in all the lesion area [19]. In patients with MS diagnosis, non-demyelinated white matter areas were taken in consideration and were identified as normal appearing white matter (NAWM) [19]. In controls, white matter areas without histopathological alterations were classified as normal white matter (NWM).

### 4.5. Descriptive and Quantitative Analyses

The distribution of GPR17^+^ cells was analyzed in each sample, overlapping the areas of interest mentioned above, identified by MOG and HLA, with the GPR17 staining. The abundance of GPR17^+^ cells in each area (ALs, CALs, CILs, and NAWMs in MS patients and NWMs in controls) was evaluated by using the following scores: ‘−’ (absence of GPR17^+^ cells), ‘+’ (low abundance of GPR17^+^ cells) or ‘++’ (high abundance of GPR17^+^ cells). The descriptive analysis included the observation of the morphology of GPR17^+^ cells.

For the quantitative analysis of GPR17^+^ cells, the areas of interest (ALs, CALs, CILs, NAWMs, NWMs) were acquired with a NanoZoomer S60 slide scanner (Hamamatsu). Positive cells were counted with the ImageJ software. Cellular density (number of GPR17^+^ cells/mm^2^) was calculated normalizing the number of GPR17^+^ cells counted in each region with the relative area (mm^2^). For this analysis, we identified one AL, CAL, CIL, and NAWM area from four different patients and one NWM area from four different healthy controls; all subjects have been randomly selected. Thus, a total of four ALs, CILs, and NWMs areas and five NAWMs areas have been studied in the quantitative analyses. In our samples, only three CALs have been identified, and all of them were selected for the quantitative analyses; however, one CAL was excluded from the study, due to the inadequate quality of the stained brain tissue, which prevented an accurate count of GPR17^+^ cells. In case of lesion, GPR17^+^ cells within 200 µm far from lesion borders have been considered (Figure S2). These extra-lesion areas alone were considered in the analysis of GPR17 expression out of lesion core (see Figure 6).

The samples analyzed for quantification were randomly selected and all the cell-counts were performed blinded to case identification.

### 4.6. Statistical Analysis

Data are presented as mean ± SEM and analyzed with GraphPad Prism 8.0 (GraphPad Software, San Diego, CA, USA). The Shapiro–Wilk normality test was used to evaluate data distribution. For all comparisons between two groups with a normal distribution, two-tailed unpaired Student’s *t*-test was performed. For multiple comparison testing, one-way analysis of variance (ANOVA) accompanied by Tukey’s post-hoc test was used. Differences were considered significant for *p*-value < 0.05. Possible outliers were verified with the Grubb’s test.

## 5. Conclusions

In conclusion, we found that: (a) GPR17 receptor is widely expressed in oligodendroglial cells in MS subjects; (b) it mainly localizes to inflamed areas of WM; (c) in ALs, GPR17 is predominantly expressed by oligodendroglial cells with rounded morphology reacting against a demyelinating damage, whereas in NAWM there is a balance between ramified and rounded morphology. In line with the previous results in animal models of disease, we propose that GPR17 may be a promising pharmacological target also in human MS, to support remyelination in the early phase of the disease and prevent myelin loss and clinical impairment.

## Figures and Tables

**Table 1:** Classification of the human specimens based on the abundance of GPR17^+^ cells. The table shows all the CASES (A) and the CONTROLS (B). In the ‘# Subjects’, all MS Tissue Bank donors are listed with their ID code. In the ‘Sample’ column, the coordinates of every autoptic brain samples which were analysed, grouped per each case or control, are indicated. In the ‘Identified conditions in WM’ the AL/CAL/CIL/NAWM/NWM which were detected in the white matter of each sample of the studied case/control are shown. The ‘GPR17 abundance’ estimated the amount of GPR17^+^ cells in the analysed condition: ‘−’ in case of absence or scarce abundance, ‘+’ for a moderate abundance and ‘++’ for a very high abundance of GPR17^+^ cells. AL: Active Lesion; CAL: Chronic Active Lesion; CIL: Chronic Inactive Lesion; NAWM: Normal Appearing White Matter in patients with Multiple Sclerosis; NWM: Normal White Matter in healthy controls.

**(A)**	**# Subjects**	**Sample**	**Identified** **Condition in WM**	**GPR17** **Abundance Score**
**CASES**	**MS 179**	P4A1	AL	+
			NAWM	++
		A3A2	AL	+
			NAWM	++
	**MS 230**	P5C2	AL	++
			NAWM	++
		P2D2	AL	++
			CIL	++
			AL	++
			NAWM	++
		P2E1	CAL	++
			AL	++
			NAWM	++
	**MS 234**	A2D4	CIL	+
			CIL	+
			AL	−
			CIL	+
			NAWM	++
	**MS 242**	P3B3	AL	++
			NAWM	++
		P4C3	NAWM	+
		A2B2	AL	++
			CIL	−
			NAWM	++
		P5A2	CIL	−
			AL	+
			NAWM	++
			NAWM	++
		P1A2	CAL	−
			AL	−
			NAWM	+
		P3A3	NAWM	++
	**MS 297**	A4B3	AL	+
			NAWM	++
		P1C2	NAWM	++
	**MS 298**	A2B4	AL	+
			NAWM	++
		P1B5	AL	+
			NAWM	++
		A4C3	CAL	++
			NAWM	++
	**MS 300**	A1A3	AL	++
			NAWM	++
		P1A2	CAL	−
			AL	−
			NAWM	+
		P3A3	NAWM	++
**(B)**	**# Subject**	**Sample**	**Identified** **Condition in WM**	**GPR17** **Abundance Score**
**CONTROLS**	**C 014**	A1D7	NWM	+
	**C 036**	A1B2	NWM	+
	**C 048**	P2F6	NWM	++
	**C 039**	A2B6	NWM	+
	**C 043**	P4D6	NWM	+

**Table 2:** Clinical and demographic data of progressive multiple sclerosis (MS) patients and healthy controls donors. SD = Standard Deviation.

Characteristics	Primary Progressive MS	Secondary Progressive MS	Total Secondary MS	Controls
Subjects, N	1	8	9	5
Age, years, mean (SD)	39 (0)	55.6 (±11.3)	53.8 (±11.9)	73.8 (±10)
Gender, women/men	1/0	5/3	6/3	1/4
Age at onset, years, mean (SD)	24 (0)	31.1 (±9)	31.2 (±8.9)	/
Time to wheelchair, median (SD)	8 (0)	7.9 (±4.4)	7.9 (±4.8)	/
Disease duration, years, mean (SD)	15 (0)	23.5 (±10.2)	22.5 (±10)	/

Globally, 29 brain samples were analyzed: 24 from MS patients and 5 from control subjects. The mean ± SD of per person analyzed sample was 2.7 ± 1 for patients with MS and 1 ± 0 for controls. Patient-level details are reported in Supplementary Table S1.

## Data Availability

The data that support the findings of this study are available from the corresponding author upon reasonable request.

## Supplementary Materials

The following are available online at https://www.mdpi.com/article/10.3390/ijms22094574/s1.

## References

[1] Podbielska M., Banik N.L., Kurowska E., Hogan E.L. (2013). Myelin Recovery in Multiple Sclerosis: The Challenge of Remyelination. Brain Sci..

[2] Starost L., Lindner M., Herold M., Xu Y.K.T., Drexler H.C.A., Heß K., Ehrlich M., Ottoboni L., Ruffini F., Stehling M. (2020). Extrinsic Immune Cell-Derived, but Not Intrinsic Oligodendroglial Factors Contribute to Oligodendroglial Differentiation Block in Multiple Sclerosis. Acta Neuropathol..

[3] Rovaris M., Confavreux C., Furlan R., Kappos L., Comi G., Filippi M. (2006). Secondary Progressive Multiple Sclerosis: Current Knowledge and Future Challenges. Lancet Neurol..

[4] Lecca D., Raffaele S., Abbracchio M.P., Fumagalli M. (2020). Regulation and Signaling of the GPR17 Receptor in Oligodendroglial Cells. Glia.

[5] Alavi M.S., Karimi G., Roohbakhsh A. (2019). The Role of Orphan G Protein-Coupled Receptors in the Pathophysiology of Multiple Sclerosis: A Review. Life Sci..

[6] Lecca D., Trincavelli M.L., Gelosa P., Sironi L., Ciana P., Fumagalli M., Villa G., Verderio C., Grumelli C., Guerrini U. (2008). The Recently Identified P2Y-like Receptor GPR17 Is a Sensor of Brain Damage and a New Target for Brain Repair. PLoS ONE.

[7] Ceruti S., Villa G., Genovese T., Mazzon E., Longhi R., Rosa P., Bramanti P., Cuzzocrea S., Abbracchio M.P. (2009). The P2Y-like Receptor GPR17 as a Sensor of Damage and a New Potential Target in Spinal Cord Injury. Brain.

[8] Chen Y., Wu H., Wang S., Koito H., Li J., Ye F., Hoang J., Escobar S.S., Gow A., Arnett H.A. (2009). The Oligodendrocyte-Specific G Protein-Coupled Receptor GPR17 Is a Cell-Intrinsic Timer of Myelination. Nat. Neurosci..

[9] Fumagalli M., Daniele S., Lecca D., Lee P.R., Parravicini C., Fields R.D., Rosa P., Antonucci F., Verderio C., Trincavelli M.L. (2011). Phenotypic Changes, Signaling Pathway, and Functional Correlates of GPR17-Expressing Neural Precursor Cells during Oligodendrocyte Differentiation. J. Biol. Chem..

[10] Fumagalli M., Lecca D., Abbracchio M.P. (2016). CNS Remyelination as a Novel Reparative Approach to Neurodegenerative Diseases: The Roles of Purinergic Signaling and the P2Y-like Receptor GPR17. Neuropharmacology.

[11] Boda E., Viganò F., Rosa P., Fumagalli M., Labat-Gest V., Tempia F., Abbracchio M.P., Dimou L., Buffo A. (2011). The GPR17 Receptor in NG2 Expressing Cells: Focus on in Vivo Cell Maturation and Participation in Acute Trauma and Chronic Damage. Glia.

[12] Ciana P., Fumagalli M., Trincavelli M.L., Verderio C., Rosa P., Lecca D., Ferrario S., Parravicini C., Capra V., Gelosa P. (2006). The Orphan Receptor GPR17 Identified as a New Dual Uracil Nucleotides/Cysteinyl-Leukotrienes Receptor. EMBO J..

[13] Bonfanti E., Bonifacino T., Raffaele S., Milanese M., Morgante E., Bonanno G., Abbracchio M.P., Fumagalli M. (2020). Abnormal Upregulation of GPR17 Receptor Contributes to Oligodendrocyte Dysfunction in SOD1 G93A Mice. Int. J. Mol. Sci..

[14] Coppolino G.T., Marangon D., Negri C., Menichetti G., Fumagalli M., Gelosa P., Dimou L., Furlan R., Lecca D., Abbracchio M.P. (2018). Differential Local Tissue Permissiveness Influences the Final Fate of GPR17-Expressing Oligodendrocyte Precursors in Two Distinct Models of Demyelination. Glia.

[15] Paladini M.S., Marangon D., Rossetti A.C., Guidi A., Coppolino G.T., Negri C., Spero V., Abbracchio M.P., Lecca D., Molteni R. (2020). Prenatal Stress Impairs Spinal Cord Oligodendrocyte Maturation via BDNF Signaling in the Experimental Autoimmune Encephalomyelitis Model of Multiple Sclerosis. Cell Mol. Neurobiol..

[16] Parravicini C., Daniele S., Palazzolo L., Trincavelli M.L., Martini C., Zaratin P., Primi R., Coppolino G., Gianazza E., Abbracchio M.P. (2016). A Promiscuous Recognition Mechanism between GPR17 and SDF-1: Molecular Insights. Cell. Signal..

[17] Franke H., Parravicini C., Lecca D., Zanier E.R., Heine C., Bremicker K., Fumagalli M., Rosa P., Longhi L., Stocchetti N. (2013). Changes of the GPR17 Receptor, a New Target for Neurorepair, in Neurons and Glial Cells in Patients with Traumatic Brain Injury. Purinergic Signal..

[18] Jäkel S., Agirre E., Mendanha Falcão A., van Bruggen D., Lee K.W., Knuesel I., Malhotra D., Ffrench-Constant C., Williams A., Castelo-Branco G. (2019). Altered Human Oligodendrocyte Heterogeneity in Multiple Sclerosis. Nature.

[19] Kuhlmann T., Ludwin S., Prat A., Antel J., Brück W., Lassmann H. (2017). An Updated Histological Classification System for Multiple Sclerosis Lesions. Acta Neuropathol..

[20] Van der Poel M., Ulas T., Mizee M.R., Hsiao C.-C., Miedema S.S.M., Adelia, Schuurman K.G., Helder B., Tas S.W., Schultze J.L. (2019). Transcriptional Profiling of Human Microglia Reveals Grey-White Matter Heterogeneity and Multiple Sclerosis-Associated Changes. Nat. Commun..

[21] Mattiace L.A., Davies P., Dickson D.W. (1990). Detection of HLA-DR on Microglia in the Human Brain Is a Function of Both Clinical and Technical Factors. Am. J. Pathol..

[22] Kutzelnigg A., Lucchinetti C.F., Stadelmann C., Brück W., Rauschka H., Bergmann M., Schmidbauer M., Parisi J.E., Lassmann H. (2005). Cortical Demyelination and Diffuse White Matter Injury in Multiple Sclerosis. Brain.

[23] Lassmann H. (2018). Multiple Sclerosis Pathology. Cold Spring Harb. Perspect. Med..

[24] Yamasaki R., Kira J.-I. (2019). Multiple Sclerosis. Adv. Exp. Med. Biol..

[25] Sensi C., Daniele S., Parravicini C., Zappelli E., Russo V., Trincavelli M.L., Martini C., Abbracchio M.P., Eberini I. (2014). Oxysterols Act as Promiscuous Ligands of Class-A GPCRs: In Silico Molecular Modeling and in Vitro Validation. Cell. Signal..

[26] Daniele S., Trincavelli M.L., Gabelloni P., Lecca D., Rosa P., Abbracchio M.P., Martini C. (2011). Agonist-Induced Desensitization/Resensitization of Human G Protein-Coupled Receptor 17: A Functional Cross-Talk between Purinergic and Cysteinyl-Leukotriene Ligands. J. Pharmacol. Exp. Ther..

[27] Wolswijk G. (2002). Oligodendrocyte Precursor Cells in the Demyelinated Multiple Sclerosis Spinal Cord. Brain.

[28] Schmidt C., Ohlemeyer C., Labrakakis C., Walter T., Kettenmann H., Schnitzer J. (1997). Analysis of Motile Oligodendrocyte Precursor Cells in Vitro and in Brain Slices. Glia.

[29] Binamé F., Sakry D., Dimou L., Jolivel V., Trotter J. (2013). NG2 Regulates Directional Migration of Oligodendrocyte Precursor Cells via Rho GTPases and Polarity Complex Proteins. J. Neurosci..

[30] Bonfanti E., Gelosa P., Fumagalli M., Dimou L., Viganò F., Tremoli E., Cimino M., Sironi L., Abbracchio M.P. (2017). The Role of Oligodendrocyte Precursor Cells Expressing the GPR17 Receptor in Brain Remodeling after Stroke. Cell Death Dis..

[31] Rone M.B., Cui Q.-L., Fang J., Wang L.-C., Zhang J., Khan D., Bedard M., Almazan G., Ludwin S.K., Jones R. (2016). Oligodendrogliopathy in Multiple Sclerosis: Low Glycolytic Metabolic Rate Promotes Oligodendrocyte Survival. J. Neurosci..

[32] Elkjaer M.L., Frisch T., Reynolds R., Kacprowski T., Burton M., Kruse T.A., Thomassen M., Baumbach J., Illes Z. (2019). Molecular Signature of Different Lesion Types in the Brain White Matter of Patients with Progressive Multiple Sclerosis. Acta Neuropathol. Commun..

[33] Hanafy K.A., Sloane J.A. (2011). Regulation of Remyelination in Multiple Sclerosis. FEBS Lett..

[34] Yeung M.S.Y., Djelloul M., Steiner E., Bernard S., Salehpour M., Possnert G., Brundin L., Frisén J. (2019). Dynamics of Oligodendrocyte Generation in Multiple Sclerosis. Nature.

[35] Calderon T.M., Eugenin E.A., Lopez L., Kumar S.S., Hesselgesser J., Raine C.S., Berman J.W. (2006). A Role for CXCL12 (SDF-1alpha) in the Pathogenesis of Multiple Sclerosis: Regulation of CXCL12 Expression in Astrocytes by Soluble Myelin Basic Protein. J. Neuroimmunol..

[36] Clarke L.E., Liddelow S.A., Chakraborty C., Münch A.E., Heiman M., Barres B.A. (2018). Normal Aging Induces A1-like Astrocyte Reactivity. Proc. Natl. Acad. Sci. USA.

[37] Raj D., Yin Z., Breur M., Doorduin J., Holtman I.R., Olah M., Mantingh-Otter I.J., Van Dam D., De Deyn P.P., den Dunnen W. (2017). Increased White Matter Inflammation in Aging-and Alzheimer’s Disease Brain. Front. Mol. Neurosci..

[38] Parravicini C., Lecca D., Marangon D., Coppolino G.T., Daniele S., Bonfanti E., Fumagalli M., Raveglia L., Martini C., Gianazza E. (2020). Development of the First in Vivo GPR17 Ligand through an Iterative Drug Discovery Pipeline: A Novel Disease-Modifying Strategy for Multiple Sclerosis. PLoS ONE.

[39] Fratangeli A., Parmigiani E., Fumagalli M., Lecca D., Benfante R., Passafaro M., Buffo A., Abbracchio M.P., Rosa P. (2013). The Regulated Expression, Intracellular Trafficking, and Membrane Recycling of the P2Y-like Receptor GPR17 in Oli-Neu Oligodendroglial Cells. J. Biol. Chem..

[40] Daniele S., Trincavelli M.L., Fumagalli M., Zappelli E., Lecca D., Bonfanti E., Campiglia P., Abbracchio M.P., Martini C. (2014). Does GRK-β Arrestin Machinery Work as a “Switch on” for GPR17-Mediated Activation of Intracellular Signaling Pathways?. Cell. Signal..

[41] Gelosa P., Bonfanti E., Castiglioni L., Delgado-Garcia J.M., Gruart A., Fontana L., Gotti M., Tremoli E., Lecca D., Fumagalli M. (2019). Improvement of Fiber Connectivity and Functional Recovery after Stroke by Montelukast, an Available and Safe Anti-Asthmatic Drug. Pharmacol. Res..

[42] Ou Z., Sun Y., Lin L., You N., Liu X., Li H., Ma Y., Cao L., Han Y., Liu M. (2016). Olig2-Targeted G-Protein-Coupled Receptor Gpr17 Regulates Oligodendrocyte Survival in Response to Lysolecithin-Induced Demyelination. J. Neurosci..

[43] De Groot C.J., Bergers E., Kamphorst W., Ravid R., Polman C.H., Barkhof F., van der Valk P. (2001). Post-Mortem MRI-Guided Sampling of Multiple Sclerosis Brain Lesions: Increased Yield of Active Demyelinating and (p)Reactive Lesions. Brain.

[44] Donninelli G., Saraf-Sinik I., Mazziotti V., Capone A., Grasso M.G., Battistini L., Reynolds R., Magliozzi R., Volpe E. (2020). Interleukin-9 Regulates Macrophage Activation in the Progressive Multiple Sclerosis Brain. J. Neuroinflamm..

[45] Magliozzi R., Howell O., Vora A., Serafini B., Nicholas R., Puopolo M., Reynolds R., Aloisi F. (2007). Meningeal B-Cell Follicles in Secondary Progressive Multiple Sclerosis Associate with Early Onset of Disease and Severe Cortical Pathology. Brain.

