# Supplementary material for: The Distribution of GPR17-Expressing Cells Correlates with White Matter Inflammation Status in Brain Tissues of Multiple Sclerosis Patients
Source: Int J Mol Sci. 2021 Apr 27;22(9):4574. doi: 10.3390/ijms22094574 (PMC8123849; doi:10.3390/ijms22094574)
Supplement: Supplementary file 1 [file ijms-22-04574-s001.zip › ijms-1191399-supplementary.pdf]

International Journal of *Molecular Sciences*

MDPI

Supplementary Materials

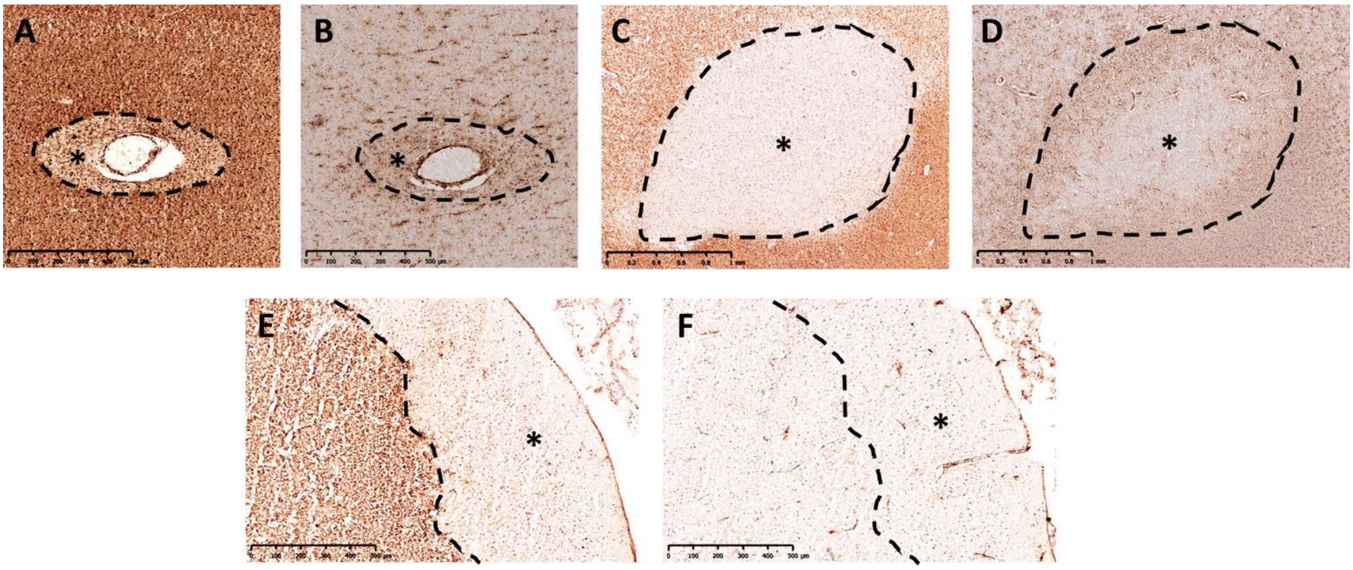

Figure S1. MS lesions with different disease activities. **A**, **C** and **E** immunochemistry for MOG**. B**, **D**, **F** immunochemistry for HLA. A and B, C and D, E and F are taken from the same lesion. All MS lesions are characterized by loss of myelin (areas not stained by MOG; dotted line and a star in the figures). A and B identify an Active Lesion (AL), where inflammatory cells are localized throughout the lesion (B). C and D identify a Chronic Active Lesion (CAL), where inflammatory cells crowded the lesion borders (D). E and F identify a Chronic Inactive Lesion (CIL) which is characterised by the absence of inflammatory cells in the lesion (F). Each MS lesion is highlighted with a dotted line and the demyelinated area is marked with an asterisk (*). Scale bar 500 μm.

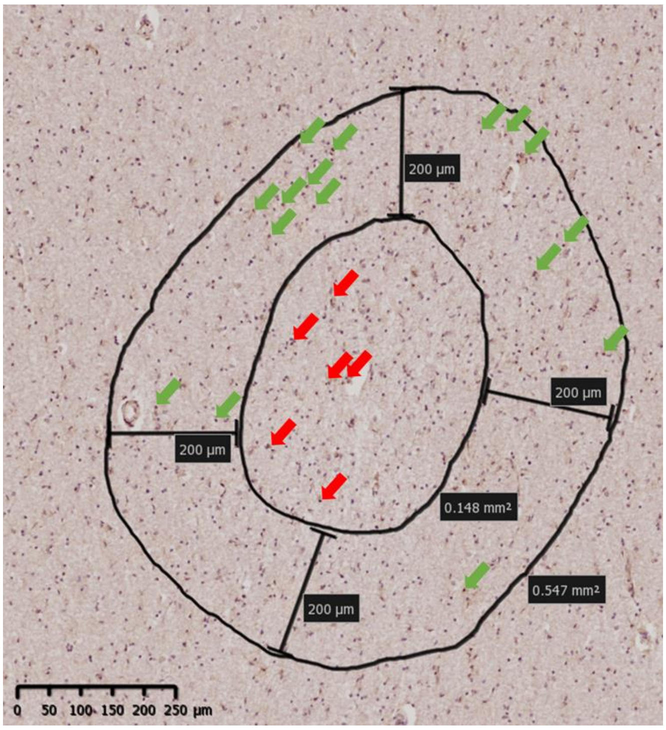

Figure S2: Representation of the partition of a demyelinating lesion we employed in order to study the spatial distribution of GPR17$^+$ cells in all the lesions we identified in the samples we took in consideration. The inner circle delimits the core of the lesion we identify by overlapping the image of the next brain slice we stained by a marker of myelin (MOG). Red arrows show the position of GPR17$^+$ cells we computed as “inside the lesion”. The external circle is fixed 200 μm far from the inner circle and it represents the external border of the lesion. The difference between the included area inside the external circle (in the micrograph= 0.547 mm^2) and the central area (in the micrograph= 0.148 mm^2) represents the border area of the lesion where we counted the GPR17$^+$ cell as posi-

tive cells outside the core lesion (green arrows). As an example an AL from MS242-P5A2 is reported, scale bar 250 μm, GPR17 staining.

Table S1: Demographic and pathological patient-level detail of Multiple Sclerosis cases and control donors.

		Patient ID Number	Age (y)	Sex	SM	Disease Duration (y)	Age of Disease Onset (y)	Post Mortem Delay (h)	Cause of Death	Time to Wheelchair (y)	Number of Analysed Samples
**Patients with MS**	**1**	MS 179	70	F	SP	26	44	20	Aspiration pneumonia, sepsis	5	2
	**2**	MS 230	42	F	SP	19	23	31	MS complications	14	3
	**3**	MS 234	39	F	RP	15	24	15	Pulmonary embolism, pneumonia.	8	1
	**4**	MS 242	57	F	SP	19	38	12	Sepsis	8	4
	**5**	MS 286	45	M	SP	16	29	7	MS complications	5	3
	**6**	MS 289	45	M	SP	18	27	9	MS complications	2	2
	**7**	MS 297	58	F	SP	13	45	8	MS complications	10	2
	**8**	MS 298	72	M	SP	43	29	11	MS complications	4	3
	**9**	MS 300	56	F	SP	34	22	13	Sepsis	15	4
**Controls**	**I**	C 014	64	M				18	Cardiac failure		1
	**II**	C 036	68	M				30	Cor pulmonale heart failure, fibrosing alveolitis, coronary artery atheroma.		1
	**III**	C 039	82	M				21	Myelodysplastic Syndrome, Rheumatoid Arthritis		1
	**IV**	C 043	87	F				12	Metastatic carcinomatosis of breast.		1
	**V**	C 048	68	M				10	Metastatic colon cancer.		1
